# Can Acute Pain Treatment Reduce Postsurgical Comorbidity after Breast Cancer Surgery? A Literature Review

**DOI:** 10.1155/2015/641508

**Published:** 2015-10-01

**Authors:** Fumimasa Amaya, Toyoshi Hosokawa, Akiko Okamoto, Megumi Matsuda, Yosuke Yamaguchi, Shunsuke Yamakita, Tetsuya Taguchi, Teiji Sawa

**Affiliations:** ^1^Department of Anesthesiology, Graduate School of Medical Science, Kyoto Prefectural University of Medicine, Kajiicho 465, Kamigyo-ku, Kyoto 6020841, Japan; ^2^Department of Pain Management and Palliative Care Medicine, Graduate School of Medical Science, Kyoto Prefectural University of Medicine, Kajiicho 465, Kamigyo-ku, Kyoto 6020841, Japan; ^3^Department of Endocrinological and Breast Surgery, Graduate School of Medical Science, Kyoto Prefectural University of Medicine, Kajiicho 465, Kamigyo-ku, Kyoto 6020841, Japan

## Abstract

Regional analgesia, opioids, and several oral analgesics are commonly used for the treatment of acute pain after breast cancer surgery. While all of these treatments can suppress the acute postsurgical pain, there is growing evidence that suggests that the postsurgical comorbidity will differ in accordance with the type of analgesic used during the surgery. Our current study reviewed the effect of analgesics used for acute pain treatments on the major comorbidities that occur after breast cancer surgery. A considerable number of clinical studies have been performed to investigate the relationship between the acute analgesic regimen and common comorbidities, including inadequate quality of recovery after the surgery, persistent postsurgical pain, and cancer recurrence. Previous studies have shown that the choice of the analgesic modality does affect the postsurgical comorbidity. In general, the use of regional analgesics has a beneficial effect on the occurrence of comorbidity. In order to determine the best analgesic choice after breast cancer surgery, prospective studies that are based on a clear definition of the comorbidity state will need to be undertaken in the future.

## 1. Background

Breast cancer surgery is a common surgical procedure performed throughout the world. American Cancer Society estimated that, in the United States, there are more than 2.8 million breast cancer survivors [1]. Acute postsurgical pain commonly exists after the breast cancer surgery. Katz et al. demonstrated that 54% of the patients who received breast cancer surgery experienced clinically meaningful pain (defined as worst pain intensity larger than or equal to 5 in 0–10 numerical rating scale) [2]. Postsurgical pain can be affected by the surgical procedure. Breast reconstruction is associated with severe and long lasting postsurgical pain [3, 4]. Abnormal sensation is less frequent in sentinel lymph node biopsy compared to the axillary lymph node dissection immediately after the surgery and up to 5 years thereafter [5].

Healthcare problems frequently seen after the breast cancer surgery include postoperative nausea and vomiting (PONV) or delayed hospital discharge during the acute phase and persistent postsurgical pain or cancer recurrence during the chronic phase [6]. The various analgesic techniques that have been reported to improve the perioperative pain conditions include regional analgesia, opioid analgesia, and perioperative medications. Moreover, there is now accumulating evidence that shows the importance of the analgesic choice in reducing the postoperative comorbidity [7]. The purpose of this study was to review the previous literature and then present the current view of the relationship between the analgesic option and perioperative comorbidity.

## 2. Quality of Recovery after the Surgery

Adequate recovery of health status early after the surgery is an important clinical endpoint. Traditionally, postsurgical recovery status has been estimated indirectly from several indices including length of hospital stay, length of recovery room stay, or PONV [8]. Recently, an established systemic scoring system, which is referred to as the “quality of recovery-40 (QOR-40)” [9], is now being used to evaluate patients who have undergone breast cancer surgery. The results of the studies investigating the relationship between analgesic modality and quality of recovery are summarized in Table 1. Regional analgesia and alpha-2 adrenergic receptor agonists have feasible effect on the quality of recovery after the breast cancer surgery.

### 2.1. Regional Analgesia

One of the most intensively investigated regional analgesia treatments in the breast cancer surgery is the paravertebral block (PVB) [2]. When combined with general anesthesia, PVB provides superior pain control after breast cancer surgery [10]. Use of a single shot PVB prior to the surgery reduces the recovery time from anesthesia and the incidence of PONV [11]. A retrospective study suggested that PVB could reduce both the length of the hospital stay and the incidence of PONV, provided the patient underwent mastectomy with immediate reconstruction [12]. Another more recently published retrospective study showed that PVB reduced opioid use and increased the fraction of patients with early discharge [13]. Beneficial effect was more prominent in patients who received immediate reconstruction. A recently published prospective study showed that, as compared to inhalational general anesthesia, ultrasound-guided PVB combined with total intravenous anesthesia using propofol resulted in a higher ambulatory surgery QoR-40 score along with a lower pain intensity, incidence of PONV [14]. Another prospective study examined the pectoral nerves (PECS) type 1 and 2 blocks that were recently developed for use as regional analgesia during breast surgery. Study findings showed that the addition of the PECS block to the general anesthesia resulted in superior analgesia, along with a lower incidence of PONV and shorter hospital stays [15].

There are inconsistencies among the previous results with regard to the efficacy of the infiltration of local anesthetics (LAs) in the wound. One study has reported that the infiltration of LAs prior to surgery did not influence the quality of the recovery [16]. Another study reported that LA infiltration after the surgery reduced both the pain intensity and the length of the hospital stay [17].

### 2.2. Systemic Analgesia

Compared to regional analgesia, fewer studies investigated the effect of systemic analgesic drugs on the recovery condition after breast cancer surgery. Preoperative oral gabapentin [18] or pregabalin [19] has been shown to reduce the pain intensity after breast cancer surgery. However, since these drugs do not reduce the incidence of PONV, this suggests that they have a smaller contribution to the quality of the recovery after the surgery. In contrast, the alpha-2 adrenergic receptor agonists, clonidine and dexmedetomidine, have been reported to have a positive effect on the quality of the recovery after surgery. Intravenous injection of clonidine [20] reduced analgesic consumption and PONV incidence, while intravenous dexmedetomidine [21] reduced both the postsurgical tramadol consumption and PONV incidence and increased the QoR-40 score after the surgery. Perioperative magnesium supplementation is associated with lower postsurgical pain and opioid consumption [22] and increased the QoR-40 after breast cancer surgery [23]. Specific treatments have been designed to reduce nausea and vomiting, reduce acute pain levels, and improve the quality of recovery after surgical procedures. Administration of dexamethasone [24] or betamethasone [25] prior to surgery has been shown to be beneficial by preventing PONV after various surgical procedures. Dexamethasone with higher dose has also been shown to reduce the postsurgical pain intensity and analgesic requirement immediately after a mastectomy [26].

Postsurgical pain should be adequately treated since that is one of physiologic disturbances of the good quality of postsurgical recovery [27]. In breast cancer surgery, regional analgesia including PVB and LA infiltration is associated with higher QoR-40 score and reduced PONV incidence and earlier discharge compared to the opioid-based analgesia. Intravenous alpha-2 adrenergic receptor agonists also increase QoR-40 score after the surgery. Occurrence and severity of PONV are closely related to the quality of the postsurgical analgesia. Both postsurgical antiemetic treatment and adequate pain control are necessary to achieve a better postsurgical recovery after breast cancer surgery.

## 3. Persistent Postsurgical Pain

Multiple investigations have attempted to identify useful treatment options for the prevention of persistent postsurgical pain after breast cancer surgeries (Table 2). Continuous local treatment with LA, single shot or continuous PVB, intravenous lidocaine, and preoperative selective serotonin-noradrenalin reuptake inhibitor successfully prevent occurrence of persistent pain after the breast cancer surgery.

### 3.1. Regional Analgesia

Two randomized controlled studies showed that while ropivacaine infiltration into the wound at the end of surgery reduced acute pain intensity, it had no effect either on the pain intensity at 2 months [28] or on the incidence of postsurgical pain between 3 and 12 months [29]. The results of these studies showed that the chronic pain incidence at 3 months after the surgery was 33 and 27% in the ropivacaine and saline group, respectively. In contrast, a repeated treatment using the eutectic mixture of local anesthetics (EMLA) started prior to the surgery and continued daily for 4 days after the surgery and reduced both the incidence and intensity of the pain at 3 months after the surgery [30]. A 50-hour continuous wound infusion of levobupivacaine administered to patients after undergoing breast cancer surgery with immediate tissue expander reconstruction reduced the incidence of persistent postsurgical pain for 3 months after the procedure [31].

A prospective study that investigated the effect of a single shot PVB at the T3 level prior to surgery found a successful reduction in the acute pain immediately after the surgery [32]. A follow-up study further demonstrated that, as compared to the saline-treated controls, PVB reduced the postsurgical pain for 12 months after the surgery during both resting conditions and when the patient was moving [33]. In another prospective study that compared the effect of PVB and repeated LA wound infiltration on persistent postsurgical pain at 12 months after the surgery, the results indicated that it was not possible to detect any superiority of the PVB versus the LA infiltration [34]. A low persistent postsurgical pain incidence was observed in both groups at 12 months after the surgery (9% for the PVB group versus 7% for the LA group).

A different prospective study investigated the effect of continuous PVB for 3 days after surgery [35]. The experimental group received 5 mL/hour of 0.4% ropivacaine whereas the control group received only saline. Both groups received a single shot PVB with 15 mL of 0.4% ropivacaine prior to the surgery. The continuous ropivacaine infusion in the experimental group reduced the pain intensity and provided better physical and emotional function at 12 months after the surgery.

### 3.2. Systemic Analgesia

Studies have shown that interventions with systemic analgesic drugs have an effect on the incidence or severity of the persistent postsurgical pain. A prospective randomized study that examined the administration of intravenous lidocaine during breast cancer surgery found that a positive effect on the persistent postsurgical pain was present after the procedure [36]. The patients in this study received a bolus injection of 1.5 mg/kg of either lidocaine or saline, with all patients at the end of the surgery, and then were administered a continuous infusion at 1.5 mg/kg/hour of the same solution initially received. Lidocaine reduced the pain intensity during movement for 4 hours after the surgery and the incidence of persistent postsurgical pain for 3 months after the surgery. A different prospective study investigated the effect of presurgical oral treatments that used either the selective serotonin-noradrenalin reuptake inhibitor (venlafaxine, 37.5 mg) or gabapentin (300 mg) [37]. Although there was a reduction in the acute postsurgical pain intensity in the gabapentin but not the venlafaxine group, there was a reduction in the persistent postsurgical pain incidence in the venlafaxine but not the gabapentin group. A previous study that administered gabapentin (1200 mg/day) or mexiletine (600 mg/day) found that symptoms associated with persistent postsurgical pain except for the burning sensation were similar between both groups and the control group [38].

In a population-based study performed in Norway, 20% of the postsurgical patients reported moderate-to-severe pain in the area of the surgery [39]. In a Canadian retrospective survey that examined patients who did not take any opioids prior to their surgery, 49.2% required an opioid prescription at time of their discharge, with 3% continuing to receive these opioids for 90 days after the surgery [40]. The prevalence of postsurgical pain has been shown to be strongly affected by the type of the surgical procedure, with the prevalence of persistent postsurgical pain highest in thoracic and breast surgery, followed by joint surgery of the knee and hip [41]. The most common factors associated with postmastectomy pain syndrome include a younger age, the type of surgical procedure (lymph node sectioning), and the presence of severe acute pain [42]. Evidences showed that continuous local treatment with LA, single shot or continuous PVB, intravenous lidocaine, and preoperative selective serotonin-noradrenalin reuptake inhibitor successfully prevent occurrence of persistent pain after the breast cancer surgery.

It is worth noting that analgesic modalities reduce acute pain and do not always prevent persistent pain after the breast cancer surgery. Future studies on the mechanisms of the chronicity of the surgical pain might be of benefit in helping to determine novel therapeutic approaches that can be used to prevent persistent pain after surgery.

## 4. Cancer Recurrence 

The stress response against the surgery impairs innate and adaptive immune function. Diminished host defense allows progressing postsurgical metastasis or recurrence of the cancer. Regional anesthesia is supposed to preserve postsurgical immune function and may improve cancer recurrence [43]. One study suggested that regional analgesia could prevent the recurrence after the breast cancer surgery [44].

One retrospective study investigated the relationship between the perioperative analgesic modality (regional analgesia with PVB or systemic morphine analgesia) and cancer survival [44]. Recurrence- and metastasis-free survival rates at 24 and 36 months were significantly higher in patients receiving PVB versus systemic morphine. As compared to patients who received sevoflurane-opioid anesthesia after the surgery, those who were administered a combination of propofol-PVB anesthesia showed reduced levels of plasma protumorigenic cytokines that included IL-1 beta, MMP3, and MMP9 [45]. Patients who received propofol-PVB exhibited increased plasma levels of IL-10, which is one of the major antitumorigenic cytokines. In vitro studies that examined patients who received general anesthesia without PVB found that the serum from these patients facilitated the proliferation of a cultured breast cancer cell line [46] and inhibited the antitumor immune activity of the natural killer T cell [47]. In another retrospective study that examined the use of different analgesics during breast cancer surgery, the cancer recurrence rate was lower when patients were treated with ketorolac [48]. The other analgesics including opioids, ketamine, and clonidine did not have any effect on the recurrence rate. The authors of this previous study suggested that the positive effect of ketorolac might be associated with an inhibitory effect on the perioperative production of prostaglandin, which is a known strong inhibitor of anticancer immunity (Table 3).

Neuroendocrine stress responses due to surgical procedures can lead to the inhibition of a patient's immune function. When combined with the inflammatory reaction that occurs after these procedures, it is possible that these surgeries might cause a patient to develop a “protumorigenic” condition [43]. Several studies have demonstrated that the postsurgical analgesic modality modifies both the surgical stress and inflammatory reaction and additionally influences the cancer recurrence or metastasis after the surgery. Data from these studies have shown that regional analgesia might increase the percentage of recurrence-free/metastasis-free patients after surgeries for gastrointestinal cancer, gynecological cancer, urological cancer, and breast cancer [49]. It should be emphasized, however, that, due to the heterogeneity of the results of these previous clinical studies, no analgesic technique has been definitively proven to increase the survival rate of a particular cancer. More definitive evidence is awaited from an ongoing prospective study investigating the effect of PVB on the cancer recurrence that will be complete in 2019 [50].

## 5. Conclusion

Not a small number of investigations about acute pain treatment after the breast cancer surgery have reported the effect of analgesic regimen on the occurrence/severity of surgical comorbidity including recovery condition, persistent postsurgical pain, and cancer recurrence. Accumulating evidence has shown that while most of the analgesic modalities can suppress the postsurgical pain intensity, not all of these analgesics can suppress the postsurgical comorbidity. Therefore, analgesic modalities need to be determined based on the feasibility of the modality achieving an effect on the postsurgical comorbidity.

## Figures and Tables

**Table 1:** Analgesic effect on the quality of recovery after the surgery.

Surgical procedure	Analgesic modality	Outcomes	Study design	Sample size	Journals (year)	1st author	Citation
Nerve block							
Mastectomy plus immediate reconstruction	PVB with Bupivacaine 0.5% versus nonblock	Reduced length of stay and PONV in PVB group	Retrospective study	*N* = 344	Ann. Surg. Oncol. (2013)	Coopey	[12]
Mastectomy with or without immediate reconstruction	PVB versus nonblock	PVB reduced PONV incidence	Retrospective study	*N* = 526	Ann. Surg. Oncol. (2014)	Fahy	[13]
Pt mastectomy with SLNB, mastectomy with or without SLNB or ALND, and mastectomy with reconstruction	PVB with ropivacaine 0.5% versus sham block	Higher QoR score, reduced discharge time, and reduced PONV in PVB group	Prospective study	*N* = 66	Anesthesiology (2014)	Abdallah	[14]
Mastectomy with ALND	PECS versus nonblock	PECS reduced PONV incidence	Prospective study	*N* = 120	Reg. Anesth. Pain Med. (2015)	Bashandy	[15]

LA injection at surgical site							
Pt mastectomy with or without ALND	Ropivacaine 0.375% versus saline before surgery	Incidence of PONV did not change between two groups	Prospective study	*N* = 60	Acta Anaesthesiol. Scand. (2000)	Johansson	[16]
Mastectomy with ALND	Bupivacaine 0.25% versus noninjection	Reduced hospital stay, pain intensity in bupivacaine group	Retrospective study	*N* = 139	Acta Chir. Belg. (2011)	Lu	[17]

Systemic analgesic drug							
Mastectomy with or without ALND, Pt mastectomy with or without ALND	Clonidine or placebo	Clonidine reduced PONV incidence	Prospective study	*N* = 68	Anesthesiology (2002)	Oddby-Muhrbeck	[20]
Mastectomy with ALND	Dexmedetomidine or saline	Higher QoR score in dexmedetomidine group	Prospective study	*N* = 92	Minerva Anestesiol. (2013)	Kim	[21]
Mastectomy with ALND	Gabapentin or placebo before surgery	Gabapentin reduced pain intensity but not PONV incidence	Prospective study	*N* = 65	Anesthesiology (2002)	Dirks	[18]
Mastectomy with or without ALND, Pt mastectomy with or without ALND	Pregabalin or placebo before surgery	Pregabalin reduced pain intensity but not PONV incidence	Prospective study	*N* = 84	Acta Anaesthesiol. Scand. (2011)	Kim	[19]
Pt mastectomy with or without ALND	Magnesium or saline	Higher QoR score in magnesium group	Prospective study	*N* = 46	Magnes. Res. (2013)	de Oliveira	[22]
Mastectomy with ALND	Dexamethasone or placebo	Incidence of PONV and intensity of pain reduced in dexamethasone group	Prospective study	*N* = 70	BMC Cancer (2010)	Gómez-Hernández	[26]
Pt mastectomy with or without ALND	Betamethasone or no-treatment control	Incidence of PONV and intensity of pain reduced in betamethasone group	Prospective study	*N* = 75	J. Clin. Anesth. (2014)	Olanders	[25]

Pt mastectomy: partial mastectomy, SLNB: sentinel lymph node biopsy, and ALND: axillary lymph node dissection.

**Table 2:** Analgesic effect on the persistent postsurgical pain.

Surgical procedure	Analgesic modality	Outcomes	Study design	Sample size	Journals (year)	1st author	Citation
Nerve block							
Pt mastectomy with or without ALND, mastectomy with or without ALND	PVB with 0.5% bupivacaine versus saline before surgery	Intensity and prevalence of pain reduced 12 mo after the surgery	Prospective study	*N* = 60	Anesth. Analg. (2006)	Kairaluoma	[33]
Pt mastectomy with ALND, mastectomy with or without ALND or SLNB	PVB with 0.5% ropivacaine before surgery versus wound injection with 0.5% ropivacaine at the end of surgery	Prevalence of pain 12 mo after the surgery was similarly low in both groups	Prospective study	*N* = 129	Ann. Surg. Oncol. (2014)	Chiu	[34]
Mastectomy with or without ALND	PVB with 0.4% ropivacaine versus saline daily for 3 days after the surgery	Prevalence of pain 12 mo after the surgery was lower in PVB group	Prospective study	*N* = 60	Ann. Surg. Oncol. (2014)	Ilfeld	[35]

LA injection at surgical site							
Mastectomy with ALND, Pt mastectomy with ALND	EMLA versus placebo for 4 days after the surgery	Pain at the surgical site reduced 3 mo after the surgery	Prospective study	*N* = 46	Reg. Anesth. Pain Med. (2000)	Fassoulaki	[30]
Mastectomy with ALND, Pt mastectomy with ALND	Ropivacaine 0.75% versus saline at the end of surgery	Similar pain condition 2 mo after the surgery	Prospective study	*N* = 46	BMC Anesthesiol. (2011)	Vigneau	[28]
Pt mastectomy with ALND, mastectomy with or without ALND or SLNB	Ropivacaine 0.375% versus saline at the end of surgery	Similar pain condition 3–12 mo after the surgery	Prospective study	*N* = 236	Anesthesiology (2013)	Albi-Feldzer	[29]
Mastectomy with reconstruction	Levobupivacaine 0.25% for 2 days after the surgery	Levobupivacaine reduced pain intensity 3 mo after the surgery	Prospective study	*N* = 60	World J. Surg. Oncol. (2014)	Strazisar	[31]

Systemic analgesic drug							
Mastectomy with ALND, Pt mastectomy with ALND	Mexiletine versus gabapentin versus placebo for 10 days	Intensity and incidence of pain 3 months after the surgery were similar. Sensation of burning pain reduced in mexiletine and gabapentin group	Prospective study	*N* = 67	Anesth. Analg. (2002)	Fassoulaki	[38]
Mastectomy with ALND, Pt mastectomy with ALND	Venlafaxine versus gabapentin versus placebo for 10 days after the surgery	Venlafaxine reduced the incidence of pain 6 mo after the surgery	Prospective study	*N* = 150	Clin. J. Pain (2010)	Amr	[37]
Pt Mastectomy with ALND or SLNB, mastectomy with ALND or SLNB	IV lidocaine versus saline during the surgery	Prevalence of pain 3 mo after the surgery reduced in lidocaine group	Prospective study	*N* = 36	Clin. J. Pain (2012)	Grigoras	[36]

Pt mastectomy: partial mastectomy, SLNB: sentinel lymph node biopsy, and ALND: axillary lymph node dissection.

**Table 3:** Analgesic effect on the cancer recurrence.

Surgical procedure	Analgesic modality	Outcomes	Study design	Sample size	Journals (year)	1st author	Citation
Mastectomy with or without ALND	PVB with 0.25% levobupivacaine versus morphine for postsurgical analgesia	Reduction of tumor recurrence and metastasis in PVB group	Retrospective study	*N* = 129	Anesthesiology (2006)	Exadaktylos	[44]
Mastectomy with ALND	Ketorolac versus sufentanil versus clonidine versus ketamine for perioperative analgesia	Ketorolac associated with reduction of tumor recurrence and metastasis	Retrospective study	*N* = 327	Anesth. Analg. (2010)	Forget	[48]

Pt mastectomy: partial mastectomy, SLNB: sentinel lymph node biopsy, and ALND: axillary lymph node dissection.
